# Supplementary material for: The interactome of intact mitochondria by cross-linking mass spectrometry provides evidence for coexisting respiratory supercomplexes
Source: Mol Cell Proteomics. 2017 Dec 8;17(2):216–32. doi: 10.1074/mcp.RA117.000470 (PMC5795388; doi:10.1074/mcp.RA117.000470)
Supplement: Supplemental Data [file supp_RA117.000470_133922_0_supp_23978_fzffwf.zip › spectra_annotation/mito_DR_spectra_annotation/291-1-92-1-9-1.pdf]

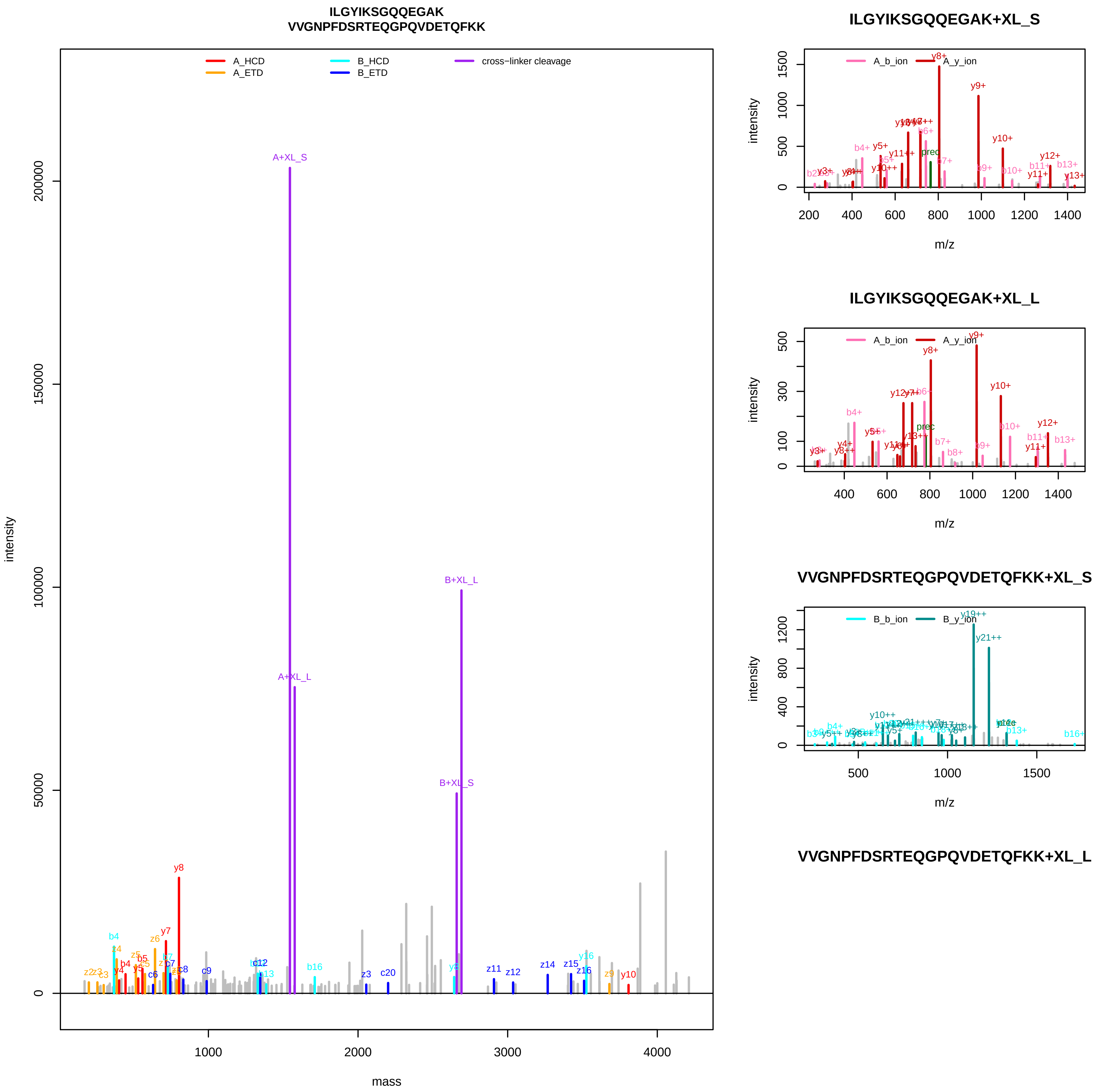

ILGYIKSGQQEGAK
VVGNPFDSRTEQGPQVDETQFKK
A_HCD
A_ETD
B_HCD
B_ETD
cross−linker cleavage
A+XL_S
A+XL_L
B+XL_L
B+XL_S
y8
y7
y10
intensity
mass
0
50000
100000
150000
200000
1000
2000
3000
4000
ILGYIKSGQQEGAK+XL_S
A_b_ion
A_y_ion
y8+
y9+
y10+
prec
intensity
m/z
ILGYIKSGQQEGAK+XL_L
A_b_ion
A_y_ion
y9+
y8+
y10+
y12+
b13+
intensity
m/z
VVGNPFDSRTEQGPQVDETQFKK+XL_S
B_b_ion
B_y_ion
y19++
y21++
y10++
b16+
intensity
m/z
VVGNPFDSRTEQGPQVDETQFKK+XL_L
